# Effect of Extraction Methods on Antioxidant Activity of Papery Skin Extracts and Fractions of Maja Cipanas Onion (*Allium cepa* L. var. *ascalonicum*)

**DOI:** 10.1155/2020/3280534

**Published:** 2020-04-16

**Authors:** Nyi M. Saptarini, Yulia Wardati

**Affiliations:** ^1^Department of Pharmaceutical Analysis and Medicinal Chemistry, Faculty of Pharmacy, Universitas Padjadjaran, Jatinangor 45363, Indonesia; ^2^Department of Pharmacy, Faculty of Mathematics and Natural Sciences, Universitas Al Ghifari, Bandung 40293, Indonesia

## Abstract

Extraction can be carried out at ambient temperature or high temperature to accelerate the extraction process of secondary metabolites from simplicia. This study aimed to determine the effectiveness of extraction methods on antioxidant activity of secondary metabolites of papery skin extracts and fractions of Maja Cipanas onion (*Allium cepa* L. var. *ascalonicum*). Extraction methods were maceration, percolation, reflux, and Soxhlet method, and then, concentrated extracts were fractionated by liquid-liquid extraction based on the polarity of secondary metabolites. Antioxidant activity was determined by the DPPH (2,2-diphenyl-1-picrylhydrazyl) method. The phytochemical screening showed that onion papery skin contained alkaloids, polyphenols, flavonoids, and tannins. The IC_50_ value of the extract, ethyl acetate fraction, and water fraction of the four extraction methods in the concentration range 25–400  *μ*g/mL were in the range of 55.62–107.08, 31.31–84.06, and 126.05–139.82  *μ*g/mL, respectively, while the IAA value was in the ranges of 0.25–0.49, 0.32–0.86, and 0.19–0.21, respectively. Variation in IC_50_ and IAA values indicate that the extraction method affects antioxidant activity, due to extracted secondary metabolites from simplicia. The highest antioxidant activity was an ethyl acetate fraction by the reflux method, while the lowest was water fraction by the percolation method.

## 1. Introduction

The production of onion (*Allium cepa* L., Liliaceae) in Indonesia was 1,470,155 tons in 2017 and increased to 1,503,436 tons in 2018, about 2.26% [1]. Onion production is fluctuating because of the imbalance of production between in-season and off-season harvest [2]. This is due to the high intensity of pest and disease if planted in out of season [3, 4].

The used part of the onion is the bulbs, while the papery skin is thrown away. The onion papery skin has not been utilized, although it contains anthocyanins [5]. Previous studies showed that Maja Cipanas onion had higher anthocyanin content than Bima Brebes onion [6] and the production of Maja Cipanas onion (10.9 tons/ha) was higher than Bima Brebes onion (9.9 tons/ha) in 2015 [1]. Anthocyanins are flavonoid derivate, which have antioxidant activity [7]. This study aimed to determine the effectiveness of extraction methods on antioxidant activity of secondary metabolites of papery skin extracts and fractions of Maja Cipanas onion (*Allium cepa* L. var. *ascalonicum*).

## 2. Materials and Methods

### 2.1. Materials

Maja Cipanas Onion (*Allium cepa* L. var. *ascalonicum*) bulbs were collected from the Gede Bage market, West Java Province, Indonesia in July 2016. Bulbs were identified by the School of Biological Sciences and Technology, Bandung Institute of Technology, Indonesia, with No. 912/II.CO2.2/PL/2016. All chemical reagents are of analytical grade (Merck, Germany), i.e., ethanol, hydrochloride acid, sodium hydroxide, n-buthanol, acetic acid, potassium chloride, potassium chloride, and sodium acetate.

### 2.2. Water Determination

Onion papery skin was peeled, washed, and dried at 40  C. Two grams of onion papery skin were dried on 105°C at an atmospheric pressure for 5  h and then weighed. Drying and weighing continued, every 1  h, until a constant weight [8].

### 2.3. Extraction

Each of 30  g onion papery skin was extracted with 300  mL of a mixture of 70% ethanol and 2 N hydrochloride acid, with a pH of 1.0 in a macerator, percolator, and reflux and Soxhlet apparatus. The extraction was repeated for three cycles with solvent replacement. Each cycle was 24  h for maceration, 8  h for percolation, and 2  h for reflux and Soxhlet method [6]. All extracts were collected and concentrated with a rotary evaporator at 40°C, and then, the yield was calculated.

### 2.4. Fractionation

The concentrated extract was dissolved in 40  mL of 60°C water and then put into a separating funnel, and 20  mL of *n*-hexane was added. The separating funnel was closed and shaken and then allowed to separate. The two fractions were separated into two different containers. The water fraction was put back into the separating funnel, and then, another 20  mL of *n*-hexane was added and carried out as above. Repeat the fractionation until uncolored *n*-hexane fraction is obtained. The *n*-hexane fraction is then held in the same container. The water fraction was placed into a separating funnel and fractioned using ethyl acetate in the same way as fractioned with *n*-hexane. Ethyl acetate and water fraction were placed in different containers and then concentrated, and the yield was calculated [9].

### 2.5. Phytochemical Screening

Fransworth method was conducted for phytochemical screening to simplicia, extracts, and fraction [10].

### 2.6. Antioxidant Activity Assay

About 40  μg/mL DPPH solution was added to 96% ethanol and allowed to stand for 20  min in a dark place. The absorbance was measured in 400–700  nm. Ascorbic acid standard, extract, and fraction were dissolved in 96% ethanol and diluted into five concentrations. Each sample (1  mL) was added to 2  ml of DPPH solution and incubated for 20  min. Absorbance was measured at maximum wavelength, and % inhibition was calculated using equation (1). The IC50 value was calculated from linear regression between % inhibition and concentration. The antioxidant activity category based on the IC_50_ value is very strong when IC_50_ < 50  *μ*g/mL, strong when IC_50_ is between 50 and 100  *μ*g/mL, moderate when IC_50_ is between 100 and 150  *μ*g/mL, and low when IC_50_ > 150  *μ*g/mL [11]. The antioxidant activity index was calculated using equation (2). The antioxidant activity category based on the AAI value is low when AAI <0.5, moderate when AAI is between 0.5 and 1.0, strong when AAI is between 1.0 and 2.0, and very strong when AAI >2.0 [12]:

(1) $$\begin{array}{c} \%\text{ inhibition}=\frac{\left(\text{A DPPH}-\text{A sample}\right)}{\text{A DPPH}}\times 100\%, \end{array}$$

(2) $$\begin{array}{c} \text{antioxidant activity index }\left(\text{AAI}\right)=\frac{\text{final DPPH concentration}\left(\mu g/mL\right)}{IC50\left(\mu g/mL\right)}. \end{array}$$

### 2.7. Statistical Analysis

Results are presented as the mean ± standard deviation (SD). One-way ANOVA followed by Student's *t*-test was used to analyze the data comparisons between groups. Values were considered statistically significant at *p* < 0.05.

## 3. Results and Discussion

### 3.1. Water Determination

Maja Cipanas onion is resistant to tuber blight due to *Botrytis allii* and potential for growing in Indonesia. A total of 5  kg of Maja Cipanas onion was produced from 36  g of dry papery skin onion with 0.72% of yield. The simplicia moisture content was 4.2 ± 0.5%, which met the requirement, i.e., less than 8%. High water content causes secondary metabolite degradation due to microorganism growth [13]. Maja Cipanas onion has pinky red papery skin [1], which suggested anthocyanins [14]. The best solvent for anthocyanin extraction is acetic acid or hydrochloric acid-contained solvent [15]. Our previous study showed that the mixture of 70% ethanol and HCl with pH 1.0 was best solvent for anthocyanin extraction [6]. All extracts of papery skin Maja Cipanas onion were red, due to an oxonium form of anthocyanin [14], with various yields and intensity because of the different extraction methods (Table 1).

The concentrated extracts were produced from cold extraction (maceration and percolation) and hot extraction (reflux and Soxhlet extraction). The efficiency of maceration and reflux extraction, i.e., the soaking method, depends on the effective diffusion and solubility of secondary metabolites [16]. The yield of maceration was lower than reflux extraction, because heating in reflux extraction increases diffusion [17]. The efficiency of percolation and Soxhlet extraction, i.e., the flowing solvent method, depends on repeated cycle extraction with a fresh solvent until all the solutes are dissolved in simplicia [18]. The yield of Soxhlet extraction was lower than percolation, due to anthocyanin instability to temperature [14]. Percolation was the extraction method with the highest yield (Table 1). This result showed that secondary metabolites can be completely extracted using flowing acidified solvents at ambient temperature.

The yield of water fractions was higher than of the ethyl acetate fractions (Table 2), due to the polarity of acidified ethanol as the extraction solvent, in which polar secondary metabolites extracted higher than nonpolar ones. The *n*-hexane fractions were not obtained because *n*-hexane is a nonpolar solvent, while the extraction solvent is polar acidified ethanol. It is suggested that no nonpolar secondary metabolites were extracted.

Alkaloids in sample solution were reacted with Dragendorff's reagent to produce an orange to orange-red precipitate, which accorded with the literature [19]. Polyphenols in sample solution were reacted with iron III chloride (FeCl_3_) to produce the green solution. These results were accorded with the literature, in which complexes of polyphenols-FeCl_3_ are blue, green, red, or purple color [20]. Tannins in sample solution were reacted with a gelatin solution containing sodium chloride to produce white precipitate, which accorded with the literature [20]. Flavonoids in sample solution were reacted with magnesium powder and concentrated hydrochloric acid to produce pink to red color. These results were accorded with the literature, in which flavonoids produce pink or red color with this reagent [21]. The phytochemical screening showed that simplicia, extracts, ethyl acetate fractions, and water fractions from the maceration, percolation, reflux, and Soxhlet method contain alkaloids, polyphenols, flavonoids, and tannins. These results show that all extraction methods can dissolve the same secondary metabolites in different quantities, which can be observed from the extract color (Table 1).

### 3.2. Antioxidant Activity Assay

**Figure 1:**
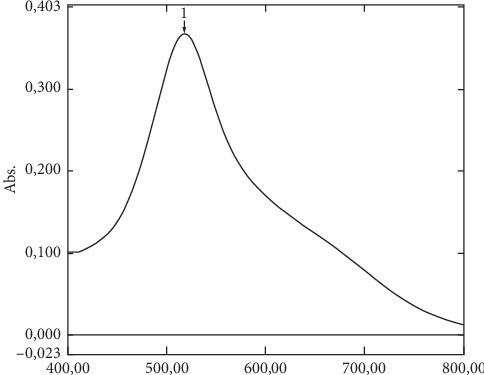
Maximum wavelength of DPPH solution. The purple DPPH solution has a maximum wavelength at 518  nm.

DPPH free radicals are organic compounds with unstable nitrogen, due to unpaired electrons [22]. DPPH solution is dark purple with a maximum wavelength of 518  nm (Figure 1). The oxidant compounds give the hydrogen atom to the DPPH free radical, which reduced to DPPH-H (1,1-diphenyl-2-picrylhydrazine) [22]. The absorbances of extract and fraction of Maja Cipanas onion were able to donate its hydrogen atoms in sufficient quantities to scavenging the DPPH free radicals (Table 3).

Three of the extracts were strong antioxidant (55.62–86.28  *μ*g/mL), except the Soxhlet extract was moderate antioxidant (107.08  *μ*g/mL). All ethyl acetate fractions were strong antioxidant (31.31–84.06  *μ*g/mL) and all water fractions were moderate antioxidant (126.05–139.82  *μ*g/mL). The AAI value was calculated with equation (2) [12], and the final DPPH concentration was 30.75  *μ*g/mL, resulting in a constant for each extract and fraction. In this work, we considered that all extracts and water fractions from all extraction methods showed low antioxidant (0.19–0.49), while all ethyl acetate fractions, except for the reflux method, were moderate antioxidant (0.64–0.86). The difference in the strength of antioxidant activity was due to differences in the content and quantity of extracting secondary metabolites in extracts and fractions. Antioxidant activity calculated by AAI is lower than the IC_50_ value, but the AAI value is more accurate, due to independent on DPPH and sample concentrations [12].

Anthocyanins have antioxidant activity [7]. In this study, total anthocyanin contents were not compared with antioxidant activity (Table 4). The total anthocyanin content of the extract was significantly different in various extraction methods (*p*=3.62 × 10^−6^). This showed that the antioxidant activity was given not only by anthocyanins, but mixed secondary metabolites in papery skin onion, i.e., alkaloids, polyphenols, flavonoids, and tannins. Alkaloids and polyphenols, including flavonoids and tannins, are the major antioxidants with antioxidant activities in natural products [23, 24].

The antioxidant activity of extracts and fractions was significantly different (*p*=9.77 × 10^−3^). The best antioxidant activity was extract from percolation (*p*=3.55 × 10^−9^), ethyl acetate fraction from the reflux method (*p*=4.78 × 10^−10^), and the water fraction from the Soxhlet method (*p*=2.41 × 10^−7^). This showed that the extraction method affects the extracted secondary metabolites from simplification, thus affecting the extracted secondary metabolites into the ethyl acetate and water fractions.

## 4. Conclusions

The extraction method affects extracted secondary metabolites, due to antioxidant activity.

## Figures and Tables

**Table 1:** The yield of extraction.

Method	Simplicia (g)	Extract (g)	Yield (%)	Color
Maceration	30	9.58	31.92	Red
Percolation	30	11.88	39.6	Dark red
Reflux method	30	9.70	32.34	Red
Soxhlet method	30	8.81	34.05	Light red

**Table 2:** The yield of fractionation.

Method	Water fraction	Ethyl acetate fraction
Maceration	67.88	23.69
Percolation	66.67	23.08
Reflux method	66.82	22.47
Soxhlet method	57.27	26.36

**Table 3:** Antioxidant activity of sample to DPPH solution (*n* = 3).

Extraction method	Sample (*n* = 3)	Concentration (*μ*g/mL)	% Scavenging	Linear equation	IC_50_ value (*μ*g/mL)	AAI
None	Ascorbic acid	2	45.51 ± 0.03	*y* = 6.52*x* + 33.00 *R*^2^ = 0.998	2.60	10.38
		4	59.12 ± 0.08			
		6	72.90 ± 0.04			
		8	85.74 ± 0.06			
		10	97.45 ± 0.07			

Maceration	Extract	25	43.86 ± 0.04	*y* = 0.083*x* + 42.86 *R*^2^ = 0.9966	86.28	0.31
		50	47.86 ± 0.05			
		100	51.17 ± 0.03			
		200	59.86 ± 0.08			
		400	75.72 ± 0.06			
	Ethyl acetate fraction	25	48.28 ± 0.06	*y* = 0.083*x* + 47.11 *R*^2^ = 0.9975	34.92	0.77
		50	52.00 ± 0.03			
		100	55.31 ± 0.04			
		200	64.00 ± 0.05			
		400	79.87 ± 0.07			
	Water fraction	25	37.10 ± 0.08	*y* = 0.106*x* + 36.04 *R*^2^ = 0.9919	131.69	0.21
		50	40.97 ± 0.04			
		100	47.86 ± 0.06			
		200	59.03 ± 0.03			
		400	77.38 ± 0.05			

Percolation	Extract	25	47.12 ± 0.05	*y* = 0.067*x* + 46.12 *R*^2^ = 0.9907	55.62	0.49
		50	50.85 ± 0.03			
		100	53.05 ± 0.06			
		200	58.47 ± 0.04			
		400	77.73 ± 0.07			
	Ethyl acetate fraction	25	49.15 ± 0.04	*y* = 0.069*x* + 47.08 *R*^2^ = 0.992	41.91	0.64
		50	50.85 ± 0.05			
		100	54.41 ± 0.08			
		200	59.32 ± 0.03			
		400	75.59 ± 0.06			
	Water fraction	25	34.07 ± 0.05	*y* = 0.129*x* + 31.95 *R*^2^ = 0.993	139.82	0.19
		50	37.46 ± 0.04			
		100	45.76 ± 0.08			
		200	60.17 ± 0.03			
		400	82.37 ± 0.07			

Reflux method	Extract	25	46.63 ± 0.03	*y* = 0.064*x* + 45.02 *R*^2^ = 0.9934	78.07	0.35
		50	49.19 ± 0.05			
		100	50.93 ± 0.04			
		200	56.74 ± 0.07			
		400	71.05 ± 0.08			
	Ethyl acetate fraction	25	73.84 ± 0.05	*y* = 0.066*x* + 47.95 *R*^2^ = 0.9923	31.31	0.86
		50	61.98 ± 0.03			
		100	53.84 ± 0.04			
		200	52.21 ± 0.08			
		400	48.72 ± 0.06			
	Water fraction	25	38.84 ± 0.08	*y* = 0.089*x* + 37.94 *R*^2^ = 0.9919	134.88	0.20
		50	42.09 ± 0.07			
		100	47.91 ± 0.04			
		200	57.33 ± 0.05			
		400	72.79 ± 0.03			

Soxhlet method	Extract	25	38.62 ± 0.09	*y* = 0.116*x* + 37.64 *R*^2^ = 0.9929	107.08	0.25
		50	45.75 ± 0.06			
		100	49.31 ± 0.03			
		200	60.57 ± 0.04			
		400	84.25 ± 0.05			
	Ethyl acetate fraction	25	45.50 ± 0.09	*y* = 0.081*x* + 43.40 *R*^2^ = 0.9955	84.06	0.32
		50	48.51 ± 0.05			
		100	51.26 ± 0.07			
		200	58.51 ± 0.04			
		400	76.44 ± 0.03			
		50	48.51 ± 0.05			
	Water fraction	25	40.69 ± 0.07	*y* = 0.086*x* + 39.13 *R*^2^ = 0.9948	126.05	0.21
		50	42.76 ± 0.04			
		100	48.51 ± 0.03			
		200	57.82 ± 0.05			
		400	73.10 ± 0.06			

**Table 4:** The IC_50_ value and anthocyanin content (*n* = 3).

Extraction method	IC_50_ value (*μ*g/mL)			Total anthocyanin content [6]
	Extract	Ethyl acetate fraction	Water fraction	
Maceration	86.28	34.92	131.69	1.181 ± 0.008
Percolation	55.62	41.91	139.82	0.597 ± 0.015
Reflux method	78.07	31.31	134.88	1.449 ± 0.013
Soxhlet method	107.08	84.06	126.05	0.342 ± 0.022

## Data Availability

The data in this study are available from the corresponding author. Anyone who needs the data in this study can ask the corresponding author by email.
